# Medical Opinion and Movement

**Published:** 1910-06-18

**Authors:** 


### Medical Opinion and Movement.

### The Osmotic Concentration of Milk.

According to the researches of Köppe and Heubner the deleterious effects of diluting cow's milk for the feeding of infants are due to the changed physical condition of the milk, and especially to the modification of its osmotic concentration. The osmotic concentration of fresh milk is fairly constant and the same as human, the freezing-point being $-56^{\circ}$ or $-57^{\circ}$ C. Milk diluted to one-third by the addition of 2 parts of an 8-per-cent. solution of lactose shows a marked diminution in osmotic concentration corresponding to a freezing-point of $-20^{\circ}$. The addition of 50 grammes of lactose per litre of milk lowers the freezing-point to $-47^{\circ}$ or $-50^{\circ}$ C. But the degree of concentration is still less than that of human milk. The addition of yet more lactose is undesirable, but the same result may be effected by the addition of 65 to 70 centigrammes of sodium chloride per litre of diluted milk. Empirically it has already been advised to make such an addition of a pinch of salt to aid the digestion of diluted milk during the early months of infant life. If the milk is diluted by the addition of an equal quantity of water it is sufficient to add 50 grammes of lactose. If ordinary sugar be used, the normal osmotic concentration can be obtained by adding 80 grammes of sugar and $1\frac{1}{2}$ gramme of sodium chloride to a litre of milk diluted to one-third. According to Dr. Engelmann this mixture gives a satisfactory food value in calories, and, although the amount of salt would appear to be a little excessive, it is well borne by healthy infants.

### Presence of Volatile Poisons in Skeletons.

Dr. Angelo de Dominicis has made an interesting contribution to the *Journal de Médicine de Paris*, in which he shows that it is possible to obtain positive evidence of a volatile poison in a corpse by chemical analysis of the bones. The presence of mineral poisons such as arsenic in the skeleton is of ancient finding, and more recently it has been shown that proof of a vegetable poison, such as strychnine, having been absorbed into the system may be obtained by analysis of the bones. The author has actually obtained such evidence in a medico-legal case of poisoning, in which 140 grammes of the bones were sufficient to furnish definite evidence of the poison. Similarly the author has obtained evidence of strychnine in the fœtal skeleton in a case of poisoning of the maternal organism by this drug. He has now carried out researches to determine the possibility of finding such a volatile poison as chloroform in the skeleton after inhalation. A dog weighing 4 kilogrammes was killed in 15 minutes by the inhalation of chloroform. It was exposed to the air for four months (May to August). The bones were then freed from the soft tissues. The long bones of the limbs were cut in pieces of 1 to 2 centimetres and reduced to powder in a bronze mortar. They were then triturated with 300 c.c. of water, 5 grammes of tartaric acid, and 2 c.c. of alcohol, and distilled. The reaction of chloroform was easily obtained in the distillate by the addition of resorcin and caustic potash. The same result was obtained with another dog after exposure for 10 months.

### Diagnosis of Tuberculous Bronchial Glands.

Dr. Michalowicz has carried out a series of observations at Vienna to determine the value of percussing the vertebral column as a means of diagnosis of tuberculous tracheo-bronchial glands in children. According to these observations, in healthy children percussion of the spine from the seventh cervical downwards gives a clear note which becomes progressively dull below the fifth or sixth dorsal vertebra. In cases of tuberculous tracheo-bronchial glands dulness is observed usually in the region of the first five dorsal vertebræ. Localised at the upper four dorsal it corresponds to the tracheal chain of glands, whereas dulness at the level of the fourth, fifth, and possibly sixth dorsal vertebra corresponds to an affection of the lower tracheo-bronchial glands. According to this author evidence of dulness during life agreed almost mathematically with the lesions found present at the autopsies. The presence of dulness, of course, only indicates the enlargement of these glands; the diagnosis of a tuberculous affection must rest upon other evidence. In one case a bronchial adenitis was found present, with corresponding dulness over the spine, but it was caused by a pneumonia following measles. Cardiac hypertrophy in no way affects the diagnostic value of spinal percussion for bronchial glands, as in such cases the dulness is localised below the eighth dorsal vertebra, and is therefore too low to be confounded with a dulness due to a bronchial adenitis.

### Abdominal Applications of Radiant Heat.

In a contribution to *Le Progrès Médical* Dr. Miramond de Laroquette, of Nancy, discusses the treatment of post-operative conditions following laparotomy—shock, intestinal paresis—by the local application of radiant heat and light to the abdomen. He thinks that by this simple means such conditions, whether appearing immediately after operation or later, may be arrested or even prevented, and he suggests that this form of treatment may also be able to combat milder forms of peritonitis by the congestive reaction which it sets up. He gives an account of several cases in which the adoption of the treatment was followed by striking and immediate results, so that from the details given one seemed fully justified in attributing the successful issue to the heat and light applications. One of the cases cited was that of a woman, aged 32, who underwent the operation of hysterectomy. The following day the condition caused anxiety: pulse 120, vomiting, micturition scanty, and no passage of flatus. Towards the evening condition became worse, with temperature 36.2° C., and pulse 140. Injections of serum and camphorated oil were made. Lavage of the stomach was followed by increased vomiting. Abdomen distended. The following day condition was not improved, and radiant heat was applied to the abdomen for 40 minutes. The pulse improved rapidly, and the patient herself felt much better. A second application two hours later was followed by the passage of flatus, and the vomiting ceased. The applications were continued at intervals, and the following day a copious stool was passed, and the patient eventually completely recovered. A special apparatus fitted with six electric lamps is used for the purpose. Experiments on animals have demonstrated that these radiant heat applications produce an intense hyperæmia of the peritoneum and mesentery with a local as well as general rise of temperature and a marked increase in peristalsis of the bowel.

### Foreign Bodies in the Stomach.

Dr. Rohmer recently reported to the Medical Society the case of a lunatic from whose stomach he had removed by gastrotomy the handles of twenty-three white-metal forks. The patient, who was employed in the kitchen, complained that for a couple of weeks he had been troubled with indigestion and abdominal pain. He admitted that he had, during a period of about six months, swallowed five or six forks unknown to the warders. Radiography showed the presence of four or five shadows in the epigastric region, which resembled those cast by the handles of forks or spoons. The foreign bodies were successfully removed, and weighed together about 14 oz. Convalescence was uneventful, and a cure resulted, the patient being none the worse for his exploit.

### Sterilisation of Water by Light.

Dr. Th. Nogier, of Lyons, has carried out a series of extensive investigations into the bactericidal action of quartz lamps of mercury vapour and their application to the sterilisation of drinking water. His experiments, reported in the *Archives d'électricité médicale*, have been made partly with Kromayer's lamp and partly with one of his own invention. He has shown that rays of small wave length possess a bactericidal action in water within a radius of 30 centimetres from the source of light, at which range an irradiation of about one minute is required for the destruction of pathogenic organisms. The effect is increased by placing the lamp in the water instead of at a distance from it. He sterilised a tank containing 26 gallons of water, tainted with colon and typhoid bacilli, in two minutes by a quartz lamp immersed in it, and working with a current of 135 volts and 9 ampères. He finds that there is no difference in the rate of growth of algæ, spirogyræ, water-plants, grasses, cress, etc., when placed in water that has been thus sterilised, as compared with that of the same placed in tap-water. Colloid substances in suspension impede or stop the process of sterilisation. The author maintains that the sole cause of sterilisation is the action of the light, and that the formation of ozone and the oxidation of the water plays no part in the process. Even the more complicated organisms are destroyed by the ultra-violet rays, for exposure to the latter will eventually kill a plant, though the effects may not be at once noticeable. He can find no marked chemical changes in the irradiated water, and believes that none take place. In discussing the industrial application of his discovery, he describes the apparatus necessary for sterilising the water-supply. He claims that with it the noxious germs and their poisons are effectively destroyed, while the water retains the salts that give it its agreeable flavour and salutary properties. Water sterilised by light in this way remains fresh and odourless.

### General Paralysis and Crime.

An interesting medico-legal question was recently discussed by Dr. Gilbert-Ballet at the Paris Academy of Medicine regarding felonious acts committed by general paralytics under the influence of an impairment of the moral sense, but before the appearance of definite symptoms pointing to the nature of the disease from which the authors of the felonies were suffering at the time of their commission. The speaker maintained that it was not sufficiently recognised that this impairment of the moral sense is frequently only revealed when the first felonious act is committed. Consequently the strangeness of the act, when the position and past record of the accused are taken into consideration, does not strike the magistrate, who, as a result, condemns the accused without having previously obtained the opinion of an expert alienist. He cited many examples, and concluded by saying that he wished to show that hereditary mental defects, or those of syphilitic origin, at times are first made manifest by the commission of the misdemeanour, and that at the moment no external sign will reveal the effect of the paralysis, which only appears at some later date.

### An Antidote for Alcohol.

Hennell, in the *Electric Medical Journal*, lays great stress on the good results which can be obtained by the use of ammonium chloride in the treatment of alcoholism in all its phases. According to the author, 30 grains of the drug dissolved in a drachm of water and given at one dose, followed by a copious draught of water, not only will counteract the effects of the alcohol and sober the patient rapidly, but will prevent the onset of delirium, and overcome the craving for alcoholic stimulants. If after the ingestion of the drug the patient has not quieted down in the course of two or three hours, some hypnotic, such as chloral hydrate or a bromide mixture, should be given. As a rule, when the patient awakes after this treatment there will be felt no craving for alcohol. The author points out that while 30 grains may seem a very large dose of ammonium chloride to give in view of the gastro-intestinal irritation which it is said to produce, when given as an antidote for alcohol only a single large dose is administered and that this is followed by a copious draught of water.

### The Age-incidence of Infantile Disorders.

Kermisson, in *Le Bulletin Médical*, maintains that each age shows a predilection for some particular disease, and has a favourite location for pathological lesions and accidents. There are two main groups of surgical affections that are constantly met with in infancy and early childhood. The first group comprises affections which mainly concern the parts of the body devoted to locomotion. The second group is made up of congenital deformities, such as spina bifida, hare-lip, cleft palate, club foot, and imperforate anus. In infants suppuration is frequently met with, especially in connection with the umbilicus, as well as erysipelas and various skin diseases. Dislocations are rare under the age of five, but may occur at the hip and wrist after that age. Fractures of the leg and arm are not common in infancy, but fracture of the femoral diaphysis occurs from slight injury. The epiphysis at this age is elastic and cartilagenous, and does not resist violence. It is in childhood and early adolescence, therefore, that most of the epiphysial injuries are seen. While intussusception is peculiar to infancy, appendicitis usually occurs later. Strangulated hernia may occur in childhood, but the obstruction in infants is spasmodic, and is reduced easily under an anæsthetic. Osteomyelitis shows a predilection for the tibia in children, whereas in infants under five years of age it is the lower end of the femur that is usually attacked. Tuberculosis is mostly confined to a single focus in infants, whereas it may have multiple foci or be generalised at a later age.

### New Sign in Rheumatism.

The rheumatism of childhood has long been notorious for its insidious onset and disastrous consequences, and clinicians have devoted much thought to its early detection as a means for successful treatment. Dr. Clemens, of St. Louis, describes in *Archives of Pediatrics* a new sign to which he attaches great importance in the rheumatism of childhood. This is enlargement of the thyroid gland, which may precede all other signs, or may be but the fourth or fifth link in the rheumatic chain; at other times he has found it to persist in association with established chronic endocarditis after all other rheumatic manifestations have disappeared. The sign is only trustworthy in children below the age of puberty, because at that epoch metabolism may undergo non-rheumatic changes which are associated with thyroid enlargement. The degree of enlargement is not great, but is sufficient to give an unnatural fulness to the neck when inspected from the front; when viewed laterally the contour of the gland is unmistakable. In the literature hitherto the relations between rheumatism and the thyroid have been practically confined to cases of exophthalmic goître. The author concludes that in any child with an enlargement of the thyroid gland a careful past history should be taken, with especial reference to growing pains, torticollis, tonsilitis, and so forth. The family history as regards rheumatism should be inquired into, and a careful physical examination of the heart should be made and signs of chorea sought for.

### The Bactericidal Power of Milk.

According to the *Revue d'Hygiène de la viande et du lait*, Dr. Bordelli, as the result of a long series of experiments, comes to the following conclusions with regard to the bactericidal power of milk: (1) This power is an obvious phenomenon which is to be observed up to a temperature of 60° C., but is not to be found between 60° and 75° C. (2) In milk heated to 60° C. the diminution in the number of germs is constantly preceded by an augmentation of their number. (3) In milk to which a filtrate of milk has been added the bactericidal power is greater than in normal milk. (4) This bactericidal power is met with in milk filtrate which has been infected. (5) The bactericidal power which is noticed in normal milk and milk filtrate cannot be attributed to the action of special bactericidal substances. (6) The progressive acidity of milk is, in the author's opinion, the principal factor in this bactericidal power. Thus the question of bactericidal power in the case of milk reduces itself to a fight for existence, a fight in which the micro-organisms of acid fermentation are the conquerors.

### Injections for Nocturnal Enuresis.

Eon, in a Paris thesis, describes the method of treating nocturnal enuresis by injections of artificial serum into the retro-rectal space. These injections act by producing a true elongation of the branches of the great sympathetic which are found in this space. The method is of easy application, and needs but simple instruments. A syringe, a platinum needle, and artificial serum are all that are required. The patient, after being anæsthetised, is placed in the left lateral position; the left limb, in contact with the bed or couch, is kept in a position of extension, the opposite thigh being moderately flexed on the pelvis and the leg on the thigh. After the usual antiseptic precautions the surgeon, standing on the right of the patient, introduces his left index finger, previously smeared with vaseline, into the patient's rectum. The site of election for inserting the needle lies about 1 centimetre in front of the coccyx, on the anococcygean raphe. The needle is inserted almost perpendicularly to the axis of the patient's body to a depth of from 8 to 10 millimetres, and is then made to turn around the coccyx and so to penetrate as far as possible into the retro-rectal space proper. Care is to be taken that the point of the needle does not pierce the rectal wall or impinge on the sacrum. The artificial serum is then introduced until the space is filled. In proportion as the cavity is occupied with the fluid will the left index finger feel the posterior rectal wall bulging forward. From 150 to 200 c.c. of fluid can be injected without ill-effects, the latter quantity, in the opinion of the author, giving the better results. In only three out of eighteen cases carefully treated has failure to effect a cure been met with. The author advises, in case of failure, a second and even a third injection. He considers that his cures are permanent, as he has had his patients under observation for a considerable period of time without a relapse occurring. Professor Jaboulay, of Lyons, was the first to describe this method of treatment.

